# Pristimerin Dampens Acetaminophen-Induced Hepatotoxicity; The Role of NF-κB/iNOS/COX-II/Cytokines, PI3K/AKT, and BAX/BCL-2/Caspase-3 Signaling Pathways

**DOI:** 10.3390/pharmaceutics17081003

**Published:** 2025-07-31

**Authors:** Mohammed A. Altowijri, Marwa E. Abdelmageed, Randa El-Gamal, Tahani Saeedi, Dina S. El-Agamy

**Affiliations:** 1 Department of Pharmacology and Toxicology, Faculty of Pharmacy, Mansoura University, Mansoura 35516, Egypt; mat-317@std.mans.edu.eg (M.A.A.); dinaagamy@mans.edu.eg (D.S.E.-A.); 2 Eradah Complex and Mental Health, Ministry of Health, Qassim 12571, Saudi Arabia; 3 Department of Medical Biochemistry and Molecular Biology, Faculty of Medicine, Mansoura University, Mansoura 35516, Egypt; drrandaelgamal@mans.edu.eg; 4 Medical Experimental Research Center (MERC), Faculty of Medicine, Mansoura University, Mansoura 35516, Egypt; 5 Department of Medical Biochemistry, Horus University—Egypt (HUE), New Damietta 44921, Egypt; 6 Department of Pharmacology and Toxicology, School of Pharmacy, Taibah University, Medina 42353, Saudi Arabia; tahsaedi@taibahu.edu.sa

**Keywords:** pristimerin, acetaminophen, NF-κB, PI3K/AKT, COX-II, caspase-3

## Abstract

**Background:** Acetaminophen (APAP) is a popular and safe pain reliever. Due to its widespread availability, it is commonly implicated in intentional or unintentional overdoses, which result in severe liver impairment. Pristimerin (Prist) is a natural triterpenoid that has potent antioxidant and anti-inflammatory properties. Our goal was to explore the protective effects of Prist against APAP-induced acute liver damage. **Method:** Mice were divided into six groups: control, Prist control, N-acetylcysteine (NAC) + APAP, APAP, and two Prist + APAP groups. Prist (0.4 and 0.8 mg/kg) was given for five days and APAP on day 5. Liver and blood samples were taken 24 h after APAP administration and submitted for different biochemical and molecular assessments. **Results**: Prist counteracted APAP-induced acute liver damage, as it decreased general liver dysfunction biomarkers, and attenuated APAP-induced histopathological lesions. Prist decreased oxidative stress and enforced hepatic antioxidants. Notably, Prist significantly reduced the genetic and protein expressions of inducible nitric oxide synthase (iNOS), cyclooxygenase-2 (COX-II), p-phosphatidylinositol-3-kinase (p-PI3K), p-protein kinase B (p-AKT), and the inflammatory cytokines: nuclear factor kappa B (NF-κB), tumor necrosis factor-α (TNF-α), and interleukins-(IL-6 and IL-1β) in hepatic tissues. Additionally, the m-RNA and protein levels of the apoptotic Bcl2-associated X protein (BAX) and caspase-3 were lowered and the anti-apoptotic B-cell leukemia/lymphoma 2 (BCL-2) was increased upon Prist administration. **Conclusion:** Prist ameliorated APAP-induced liver injury in mice via its potent anti-inflammatory/antioxidative and anti-apoptotic activities. These effects were mediated through modulation of NF-κB/iNOS/COX-II/cytokines, PI3K/AKT, and BAX/BCL-2/caspase-3 signaling pathways.

## 1. Introduction

Acetaminophen (Paracetamol, APAP) is a commonly used antipyretic medication that has been proven to induce liver damage when taken in excessive amounts beyond the recommended dosage. APAP overdose, whether deliberate or unintentional, is the primary cause of hepatotoxicity in the United States and continues to be a global problem. Until now, N-acetyl cysteine (NAC) has been the antidote for APAP-induced toxicity, although it has a rather limited therapeutic range in many patients. Hence, there is a continuous need for novel treatment intervention for APAP hepatotoxicity [1,2]. Since the 1970s, the mouse model of APAP hepatotoxicity has been widely employed because, in terms of hepatic injury and recovery, it most closely resembles the human pathophysiology [3,4]. The basic processes of APAP-induced liver damage in mice have been verified in patients who have overdosed on APAP and in human liver cells, and the degree of liver damage is quite comparable in both mice and humans [4,5]. Nevertheless, the rate of injury advancement is significantly higher in mice compared to humans, as in mice, the highest alanine aminotransferase (ALT) values, which serve as a sign of the death of liver cells, occur within 12 to 24 h [6,7].

APAP undergoes metabolic activation in the liver through the action of cytochrome P450, resulting in the formation of N-acetyl-p-benzoquinone imine (NAPQI), which is a reactive metabolite that forms covalent bonds with different cellular proteins [5]. NAPQI disrupts complex I/II of the mitochondrial electron transport chain that results in the formation of excessive superoxide radicals, which interfere with different cellular biological processes initiating oxidative stress, a crucial process that controls the development of APAP-induced hepatic injury [8]. There are many pathogenic pathways that control the magnitude of APAP-induced hepatic injury. The PI3K/AKT signaling pathway possesses a unique function in protecting the liver by controlling cell death in the presence of oxidative damage and inflammation [9]. The PI3K/AKT signaling pathway is one of the main targets for hepatic protection as AKT has the ability to control intracellular glucose metabolism, allowing cells to adjust to variations in their surroundings and improve cell survival [10]. On the other hand, ROS production is directly engaged in the modulation of multiple signaling molecules, including PI3K [11]. The rise in intracellular ROS and resultant oxidative stress prompts the compensatory stimulation of the PI3K/AKT signaling pathway [12], and this process helps to reduce oxidative stress injury. NF-κB is an important transcriptional regulator of the inflammatory response in the liver. It has been shown that NF-κB is a cornerstone in almost all types of liver diseases, like viral hepatitis, alcoholic liver disease, and biliary liver disorders [13]. The generation of pro-inflammatory cytokines and chemokines by injured tissue relies on the activation of NF-κB [14]. Therefore, NF-κB can represent a potential target for the treatment of hepatocellular damage caused by an overdose of APAP [15,16,17].

Pristimerin (Prist), is the main active constituent found in various plants belonging to the Celastraceae family [18]. This triterpenoid molecule has significant antioxidant and anti-inflammatory activities [19]. Prist exhibited potent anti-inflammatory efficacy by preventing the generation of different types of cytokines, and this mechanism is associated with the downregulation of inflammatory pathways such as NF-κB and MAPK [20,21]. Prist has strong therapeutic effectiveness in treating autoimmune arthritis in rats [22,23]. Additionally, Prist exhibits anticancer and antiproliferative properties that are attributed to its ability to block NF-κB activation in tumor cell proliferation [18], and to inhibit proteasome activity, tumor cell motility, and angiogenesis [22]. Interestingly, Prist has shown hepatoprotective activity against autoimmune hepatitis, through the activation of the Nrf2/HO-1 pathway and downregulation of NF-κB-triggered inflammation cascade [24]. However, Prist’s ability to prevent hepatic damage caused by APAP has not been addressed before. Hence, the current study aimed to determine whether Prist could have a beneficial impact in the case of APAP-induced hepatic damage and examine the potential underlying mechanisms that could contribute to these effects.

## 2. Materials and Methods

### 2.1. Animals and Study Design

Male adult BALB/c mice, with an average weight of 35–40 g, were obtained from the Egyptian Organization for Biological Products and Vaccines, Giza, Egypt. For one week prior and during the experiment, animals were housed under standard conditions: cages, temperature, humidity, dark and light cycle, unrestricted food pellets, and tap water. The experimental design was approved by the Research Ethics Committee, Faculty of Pharmacy, Mansoura University, Egypt, code number: MU-ACUC (PHARMA.MS.23.07.19). Animal care and experimentation were conducted according to the National Institutes of Health Guide for the Care and Use of Laboratory Animals (NIH publication no. 85-23, revised 2011).

### 2.2. Drugs and Chemicals

APAP and Prist (Cat No. HY-N1937) were obtained as pure powders from (Sigma, MO, USA) and (MedChemExpress LLC, 1 Deer Park Dr, Suite Q, Monmouth Junction, NJ 08852, USA NJ, USA, respectively). N-acetylcysteine (NAC) was obtained as a pharmaceutical formulation (Fluimucil, The Cathay Drug Company Inc., Makati, Philippines). Thiopental was obtained as a pharmaceutical formulation (EIPICO company, Tenth of Ramadan City, Egypt). All other chemicals employed in this investigation were obtained from the El-Nasr Chemicals Company (Abou-Zaabal, Cairo, Egypt) and are of fine analytical grade (ACS/Reagent Grade).

Mice were treated as follows after being randomly assigned to six groups of six animals: control group; Prist control group where animals received Prist (0.8 mg/kg) intraperitoneally (*i.p.*) for 5 days [24,25,26]; APAP group where animals received APAP (400 mg/kg) *i.p.* [27]; APAP + NAC group where animals received APAP (400 mg/kg) and after 2 h, NAC (400 mg/kg) *i.p.* [28,29]; and two Prist + APAP groups where mice received Prist at two different doses (0.4 or 0.8 mg/kg) *i.p.* for 5 days and APAP (400 mg/kg) *i.p.* on day 5, one hour after Prist intake.

Twenty-four hours after APAP administration, the mice were anesthetized using thiopental (70 mg/kg, *i.p.*); then, blood samples were taken using capillary tubes from the medial retro-orbital venous plexus. Subsequently, the blood underwent centrifugation (3000 rpm for 15 min); then, the serum was gathered and stored at −80 °C for further analysis.

The hepatic tissues were dissected, and the left median lobe was homogenized in cold phosphate-buffered saline (PBS) solution. The homogenate was subjected to centrifugation (10,000 rpm for 20 min at 4 °C) and the obtained supernatant was carefully gathered and stored in a temperature of −80 °C for subsequent biochemical determinations. The right median lobe of the hepatic tissue was preserved in a 10% (*v*/*v*) solution of neutral-buffered formalin for histopathological and immunohistochemical evaluation. Another 2 sections of the left median lobe were rapidly frozen in liquid nitrogen and preserved at −80 °C for quantitative RT-PCR and Western blot analysis.

### 2.3. Histopathology

Liver samples were dehydrated in increasing alcohol grades, cleaned with xylene, and then embedded in paraffin wax. The embedded samples were cut with microtome at 5 μm thick sections. The section was stained with hematoxylin and eosin (H and E), and then examined under the light microscope (Olympus CH2, Japan) [30]. The histopathological evaluation was performed and the hepatic tissue scoring for lesions severity was conducted as follows: 0 for absent; 1 for mild; 2 for moderate; and 3 for severe as shown in Table 1 [31]. A basic histopathological scoring for lesions was conducted as previously mentioned, with little modification [32].

### 2.4. Ultra-Structure Examination of Liver by Electron Microscope

Using a specialized razor, the hepatic tissues were cut into pieces for fixation, then washed with PBS solution and fixed in 1% osmium tetrachloride. Tissues were dehydrated using gradual increasing concentrations of alcohol, embedded into an epoxy resin capsule, and stained with toluidine blue to select the appropriate area. Then, ultrathin sections were cut using a diamond knife on an ultramicrotome. Sections were put on copper grids and stained with uranyl acetate followed by lead citrate [33,34]. An electron microscope JEOL_JEM-2100 (Jeol, Tokyo, Japan) was used to take pictures of the liver tissue.

### 2.5. Immunohistochemical Examination

The expression levels of COX-II, NF-κB p65, TNF-α, BAX, BCL-2, and caspase-3 in hepatic tissues were determined by immunostaining utilizing the Avidin-Biotin Complex method [35]. The following antibodies were recruited: COX-II (Cat #GB11077-1, DiagnoCine, Totowa, NJ, USA), NF-κB-p65 (Cat #PA5-29342, Thermo Fisher Scientific Anatomical Pathology, CA, USA), TNF-α (Cat #GB11188, Wuhan Servicebio Technology Co., Ltd., Wuhan, China), BCL-2 (Cat #61-0005, Genemed, CA, USA,), BAX (Cat #A12009, Cummings Park, Ste. Woburn, MA, USA), and Caspase-3 (Cat #GB11532, Wuhan Servicebio Technology Co., Ltd., Wuhan, China). For quantification of IHC-positive cells, the whole tissue sections with high-resolution images were entered in ImageJ software. A virtual dissection, or extracting the ROI (region of interest), in whole tissue sections at magnifications up to 40× were performed to estimate relative antigen content. The digital image was processed using the color deconvolution method to separate the brown DAB chromogen from the hematoxylin counterstain on a microscope slide. A monochrome image representing the DAB content was then subjected to frequency analysis using ImageJ software version 1.54 (FIJI, National Institutes of Health, Bethesda, MD, USA), and calculation of the area fraction of DAB (antigen) was performed [36]. The result was expressed as a percentage of immunopositive-stained area in each hepatic section and expressed as mean ± standard deviation.

### 2.6. Liver Function Biomarkers

Albumin was determined using a kit obtained from Spectrum Diagnostics (Cat #211001, Cairo, Egypt), while the levels of alanine aminotransaminase (ALT), aspartate aminotransaminase (AST), alkaline phosphatase (ALP), and gamma-glutamyl transpeptidase (GGT) were determined using kits obtained from Liquicheck, Agappe Hills, Dist. Ernakulam, Kerala, India (Cat #11409003, 11408003, 11401009, and 11416005, respectively), following the manufacturer’s instructions.

### 2.7. Oxidative Status Biomarkers

The concentration of reduced glutathione (GSH), glutathione reductase (GR), glutathione-S-transferase (GST), total antioxidant capacity (TAC), superoxide dismutase (SOD), and malondialdehyde (MDA) was determined in the hepatic tissue homogenates using commercially available kits from Bio-diagnostic, Giza, Egypt (Cat #GR 25 11, GR 25 23, GT 25 19, TA 25 13, SD 25 21, and MD 25 29, respectively).

### 2.8. Real-Time Reverse Transcription PCR (qRT-PCR)

To extract total cellular RNA, QIAzol reagent (Qiagen, Hilden, Germany) was utilized and Thermo Scientific NanoDrop One (Wilmington, DE, USA) examined the yield of RNA to calculate its concentration and purity. First-strand cDNA was synthesized from 1 µg of RNA using a Proflex Thermal Cycler (Applied Biosystems, Foster City, CA 94404, USA) and a cDNA synthesis kit (Applied Biotechnology, Abbasya, Cairo, Egypt). The primers were subjected to 30 min of reverse transcription at 42 °C, followed by 5 min for terminating the reaction at 70 °C.

Azure Cielo 6 (Azure, San Antonio, TX, USA) real-time PCR equipment was used to amplify cDNA templates. The 20 μL reaction volume was made up of 10 µL of Willowfort SYBR green PCR Master Mix (Willowfort, Nottinghamshire, NG1 1GF, UK), 1 µL of cDNA template, 2 µL (10 pmol/μL) of gene primer, and 7 µL of nuclease-free water. After adjusting the thermal profile for 2 min at 95 °C, there were 40 cycles: 5 s of denaturation at 95 °C, followed by 30 s of annealing and extending at 60 °C.

Some primers were newly designed using Primer3 Input (version 0.4.0), others were extracted from previous publications as demonstrated in the following table. Glyceraldehyde-3-phosphate dehydrogenase (GAPDH) was used as a reference gene. The Primer-BLAST program was used to check all the primers’ specificity. For verification of the PCR products’ specificity, a melting curve analysis was performed. From Vivantis (Vivantis Technologies, Shah Alam, Selangor, Malaysia), the primer sets were acquired. Samples were run in triplicates. Relative gene expression levels were represented as ΔCt = Ct _target gene_ − Ct _housekeeping gene_; and to calculate the fold change in gene expression, the 2^−ΔΔCT^ method was utilized [37]. **Gene****Sequence at Annealing Temperature (60 °C)****Product Size (bp)****RefSeq****References**iNOSF: CAGCTGGGCTGTACAAACCTT
R: CATTGGAAGTGAAGCGTTTCG95NM_001313921.1[38]COX-IIF: ACACACTCTATCACTGGCACC
R: TTCAGGGAGAAGCGTTTGC274NM_011198.5[39]TNF-αF: TGAACTTCGGGGTGATCGGT
R: GGTGGTTTGTGAGTGTGAGGG99NM_001278601.1[40]IL-6F: TACCACTTCACAAGTCGGAGGC
R: CTGCAAGTGCATCATCGTTGTTC116NM_001314054.1[41]IL-1βF: GCAACTGTTCCTGAACTCAACT
R: GGGTCCGTCAACTTCAAAGA81NM_008361.4[40]BCL-2F: CCTGTGGATGACTGAGTACCTG
R: AGCCAGGAGAAATCAAACAGAGG123NM_009741.5[40]BAXF: TGAAGACAGGGGCCTTTTTG
R: AATTCGCCGGAGACACTCG140NM_007527.3[40]

### 2.9. Western Blot Analysis

#### Protein Extraction Procedure

The ReadyPrepTM protein extraction kit (total protein) provided by Bio-Rad Inc (Catalog #163-2086) was added to each sample of homogenized tissue according to manufacturer guidelines. To determine protein concentration in each sample, the Bradford Protein Assay Kit (SK3041) for quantitative protein analysis was provided by Bio basic Inc (Markham, York Region, ON, Canada) and performed according to manufacture guidelines. Then, 20 μg of protein concentration from each sample was loaded with an equal volume of 2× Laemmli sample buffer containing 10% 2-mercaptoehtanol, 0.004% bromophenol blue, 4% sodium dodecyl sulfate, 20% glycerol, and 0.125 M Tris HCl; and pH was optimized to 6.8. Then, the mixture was boiled at 95 °C for 5 min to guarantee protein denaturation before loading on polyacrylamide gel electrophoresis.

Samples were separated on polyacrylamide gel electrophoresis. The separated proteins were then transferred to polyvinylidene fluoride membranes. Prior to overnight incubation at 4 °C, the membranes were blocked with 5% bovine serum albumin for 1.5 h. Primary antibodies of β-actin, p-PI3K, t-PI3K, p-AKT, and t-AKT were incubated with a horseradish peroxidase (HRP)-conjugated goat anti-rabbit/mouse polyclonal secondary antibody (dilution ratio of 1:10,000, ASPEN) for a duration of 2 h at ambient temperature. The chemiluminescent signals were determined by a CCD camera-based imager, and image analysis was conducted using ChemiDoc MP image analysis software version 3.0.1 and presented as the target protein/control protein ratio.

### 2.10. Determination of Inflammatory and Apoptotic Markers by ELISA

iNOS, COX-II, NF-κB, TNF-α, IL-6, B-cell lymphoma 2 (BCL-2), BCL-2-Associated X-protein (BAX), and caspase-3 proteins concentrations were determined using standard commercial kits (iNOS: Cat #NBP2-80256, Novus Biologicals, centennial, USA, NF-κB: Cat #EM1230, FineTest, Wuhan, China, TNF-α: Cat #SEA133Mu, CLOUD-CLONE CORP, Katy, TX 77494, USA, IL-6: Cat #431301, BioLegend Way, San Diego, CA, USA, COX-II: Cat #E0605Mo, BTLAB, Shanghai, China, BCL-2: Cat #NBP2-69946, Novus Biologicals, centennial, USABAX: Cat #E4512-100, Biovision, Milpitas, CA, USA, and caspase-3: Cat #E4591-100, Biovision, Milpitas, CA, USA, respectively), which utilized sandwich enzyme-linked immunosorbent assay (ELISA) technology.

### 2.11. Statistical Analysis

The quantitative data were presented as mean ± standard deviation (SD). Statistical significance was defined as a *p* value of less than 0.05. The two-tailed unpaired Student’s *t*-test or analysis of variance (ANOVA), followed by Tukey’s post hoc test was used to compare the groups. The Kruskal–Wallis test, followed by Dunn’s test, was utilized for non-parametric data, and data was expressed as median ± interquartile range (IQR). Normality of data was tested using the “Shapiro–Wilk normality test” in Graphpad software Prism V 9 (GraphPad Software Inc., San Diego, CA, USA).

## 3. Results

There was no significant difference between the control and Prist groups in all the measured parameters.

### 3.1. Prist Improved APAP-Induced Hepatic Histopathological Lesions

**Figure 1:**
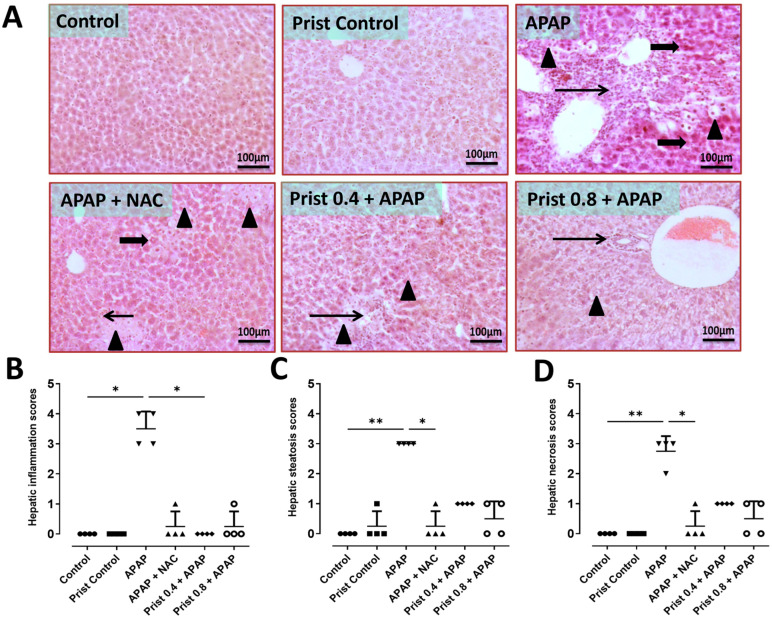
The effect of Prist on APAP-induced histopathological changes using H and E staining. Low magnifications: ×100 bar 100. (**A**) Histological sections of mice liver; control group showed normal histological appearance of hepatocytes; Prist control group showed normal architecture of up to 90% of hepatic parenchyma; APAP-injected group showed severe hepatitis characterized by coalescing extensive replacement of hepatic parenchyma with high numbers of macrophages, lymphocytes, and plasma cells extending and separating either shrunken, hyperesinophilic with pyknotic nucleus (necrosis), or ballooned, vacuolated hepatocytes (degeneration); (APAP + NAC) group showed multifocal hepatocellular steatosis with few inflammatory aggregates and few hepatocellular necrosis; (Prist 0.4 + APAP) group showed focal aggregations of cellular infiltrates admixed with mild steatotic hepatocytes; (Prist 0.8 + APAP) group showed focal periportal aggregations of low numbers of inflammatory cells. The remaining hepatocytes showed moderate hepatocellular steatosis. (**B**) Scores of inflammation; (**C**) scores of steatosis; (**D**) scores of necrosis. Thick arrow = hepatocellular necrosis, thin arrow = inflammation, arrowhead = hepatocellular steatosis, and twisted arrow = hemorrhage. Data are expressed as median ± interquartile range (*n* = 4). APAP: acetaminophen; NAC: N-acetyl cysteine; Prist: Pristimerin. * *p* < 0.05, ** *p* < 0.01 using Kruskal–Wallis, followed by Dunn’s multiple comparison test.

As shown in Figure 1A, H and E-stained hepatic specimens of the control and Prist groups showed normal histological appearance of hepatocytes. On the other hand, APAP-intoxicated mice exhibited severe hepatitis characterized by coalescing extensive replacement of hepatic parenchyma with high numbers of macrophages, lymphocytes, and plasma cells extending and separating either shrunken, hyperesinophilic with pyknotic nucleus (necrosis), or ballooned, vacuolated hepatocytes (degeneration). However, the hepatic parenchyma of NAC-pretreated group showed multifocal hepatocellular steatosis with few inflammatory aggregates and few hepatocellular necrosis. Interestingly, the Prist 0.4-pretreated group showed focal aggregations of cellular infiltrates admixed with mild steatotic hepatocytes and Prist 0.8-pretreated group showed focal periportal aggregations of low numbers of inflammatory cells. The remaining hepatocytes showed moderate hepatocellular steatosis (Figure 1B), steatosis (Figure 1C), and necrosis (Figure 1D) showed a significant elevation in these scores in APAP group compared to the control group. However, NAC and Prist pretreatment succeeded to mitigate the evolution of APAP-induced lesions and significantly improved all the pathogenic changes listed above.

**Figure 2:**
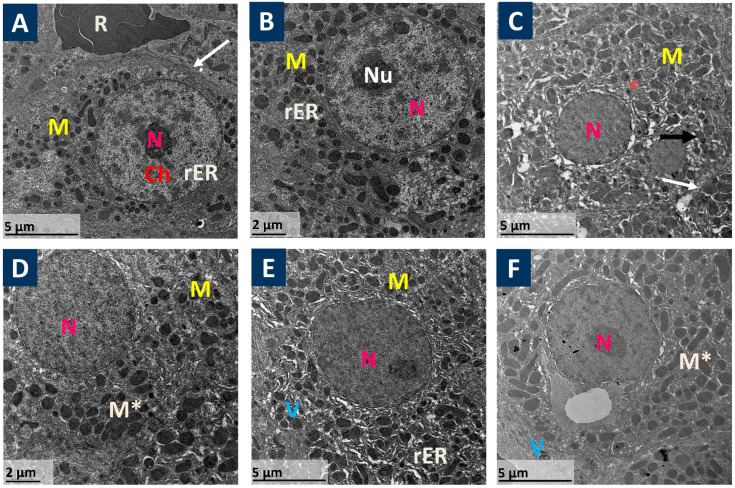
The effect of Prist on APAP-induced histopathological changes using transmission electron micrographs (TEMs). (**A**,**B**) Control and Prist control groups showed active normal hepatocytes; the nuclei (N) are large, round, euchromatic, and with regular outline. The heterochromatin (Ch) is normally distributed as scattered clumps in the nucleoplasm and as a band under the nuclear envelope; numerous normal mitochondria (M) are distributed normally in the cytoplasm, and normal blood sinusoids filled with some red blood cells (R). Also, a normal cell junction (arrow), in addition to profiles of rough endoplasmic reticulum rER and lysosomes (L), was observed. (**C**) The APAP-injected group showed apoptotic hepatocytes with pyknotic nuclei (N), cytoplasmic damaged materials (asterisk), vacuoles (V), and swollen pleomorphic mitochondria (m). Abnormal plasma membrane (Black arrow) and abnormal cell junction (white arrow). (**D**) (APAP + NAC): Hepatocytes retained its normal shape with a round nucleus; the cytoplasm contains numerous normal mitochondria (M) and some swollen mitochondria (M *). (**E**) (Prist 0.4 + APAP): Hepatocytes lost their architecture, with irregular nuclei (N), abnormal cellular components, and dilated endoplasmic reticulum cisternae (white arrow). (**F**) (Prist 0.8 + APAP): Hepatocytes appear healthy, but the cytoplasm contains numerous swollen mitochondria (M *).

As shown in Figure 2, transmission electron micrographs of mice hepatic sections revealed that the control and Prist groups showed active, normal hepatocytes with large and round nuclei, euchromatic and with regular outlines. The heterochromatin was normally distributed as scattered clumps in the nucleoplasm and as a band under the nuclear envelope; numerous normal mitochondria were distributed normally in the cytoplasm, and normal blood sinusoids were filled with some red blood cells. Also, a normal cell junction, in addition to profiles of rough endoplasmic reticulum and lysosomes, was observed (Figure 2A,B). Meanwhile, the APAP group showed apoptotic hepatocytes with pyknotic nuclei, cytoplasmic damaged materials and vacuoles, swollen pleomorphic mitochondria, abnormal plasma membranes, and abnormal cell junction (Figure 2C). On the other hand, the NAC-treated group showed that hepatocytes retained their normal shape with round nucleus, and the cytoplasm contained numerous normal mitochondria and some swollen mitochondria (Figure 2D). In addition, the Prist 0.4-pretreated group showed that hepatocytes lost their architecture, with irregular nuclei and abnormal cellular components, and displayed dilated endoplasmic reticulum cisternae (Figure 2E). The Prist 0.8-pretreated group showed that hepatocytes appeared healthy, although the cytoplasm contained numerous swollen mitochondria (Figure 2F).

### 3.2. Prist Ameliorated Serum Biomarkers of APAP-Induced Hepatic Intoxication

**Figure 3:**
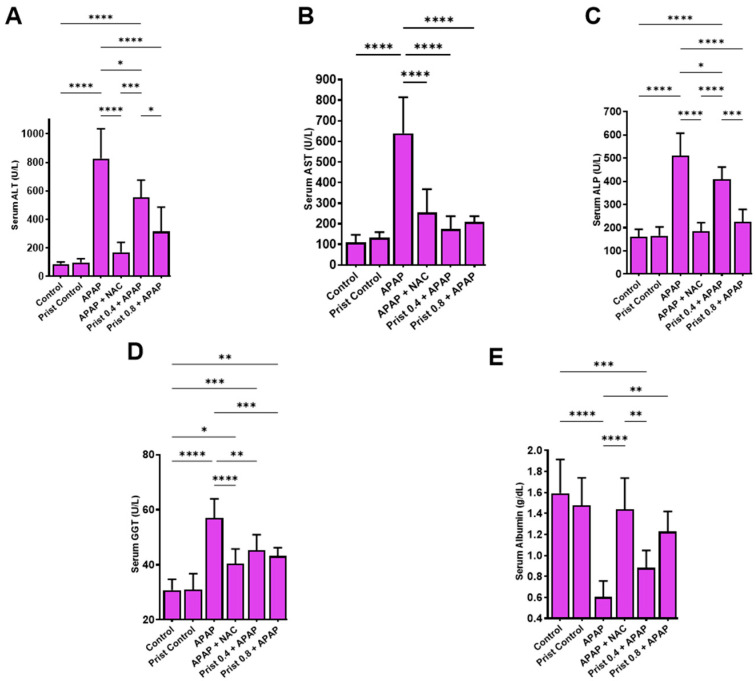
Effect of Prist on liver function biomarkers. (**A**) ALT: alanine aminotransferase; (**B**) AST: aspartate aminotransferase; (**C**) ALP: alkaline phosphatase; (**D**) GGT: gamma-glutamyl transferase; (**E**) albumin. Data are expressed as mean ± SD (*n* = 6). APAP: acetaminophen; NAC: N-acetyl cysteine; Prist: Pristimerin. * *p* < 0.05, ** *p* < 0.01, *** *p* < 0.001, and **** *p* < 0.0001 using one-way ANOVA followed by the Tukey–Kramer multiple comparisons post hoc test.

Serum ALT, AST, ALP, and GGT levels were raised considerably (*p* < 0.0001) by APAP administration, while serum albumin levels were markedly lowered (*p* < 0.0001) as compared to the control mice (Figure 3A–E, respectively). This effect was significantly mitigated in Prist-pretreated animals in comparison with the APAP group. The Prist 0.8-pretreated mice showed a significant effect as compared to Pris 0.4-pretreated one. Noteworthy, the effect of Prist (0.8 mg/kg) was nearly equivalent to that of the standard antidote, NAC, which caused a marked (*p* < 0.05) reduction in serum ALT, AST, ALP, and GGT levels, and an elevation in serum albumin.

### 3.3. Prist Augmented Hepatic Antioxidants and Counteracted APAP-Induced Hepatic Oxidative Stress

**Figure 4:**
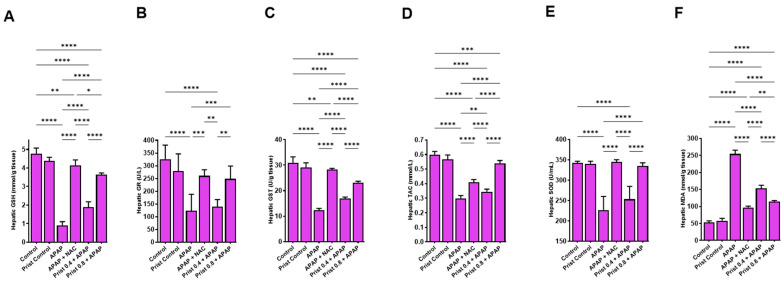
Impact of Prist on oxidative stress in liver tissue. (**A**) GSH: reduced glutathione; (**B**) GR: glutathione reductase; (**C**) GST: glutathione-S-transferase; (**D**) TAC: total antioxidant capacity; (**E**) SOD: superoxide dismutase; (**F**) MDA: malondialdehyde. Data are expressed as mean ± SD (*n* = 6). APAP: acetaminophen; NAC: N-acetyl cysteine; Prist: Pristimerin. * *p* < 0.05, ** *p* < 0.01, *** *p* < 0.001, and **** *p* < 0.0001 using one-way ANOVA followed by the Tukey–Kramer multiple comparisons post hoc test.

A single injection of APAP resulted in a marked reduction in hepatic antioxidants such as GSH, GR, GST, TAC, and SOD (Figure 4A–E), and markedly (*p* < 0.0001) elevated the hepatic levels of the lipid peroxidative marker, MDA (Figure 4F), when compared to control mice. Prist (0.8 mg/kg) pretreatment alleviated these alterations and markedly elevated the lowered levels of GSH, GR, GST, TAC, and SOD; it also significantly lowered (*p* < 0.05) hepatic MDA content as compared to the APAP-injected group. Administration of the standard antidote NAC markedly restored the hepatic levels of the previous parameters to near baseline levels when compared to the APAP group.

### 3.4. Prist Attenuated Hepatic iNOS and COX-II Expression

**Figure 5:**
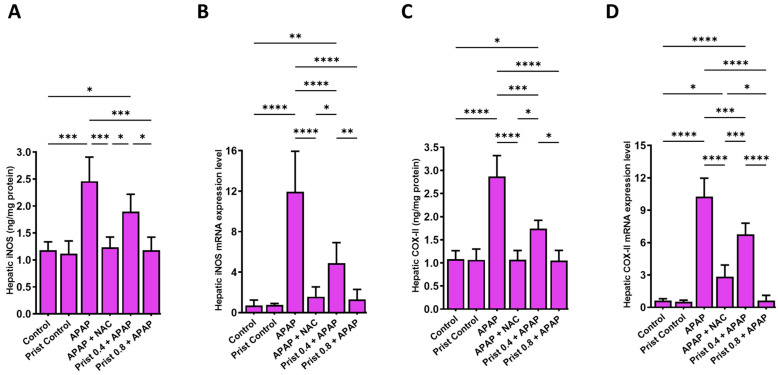
Effect of Prist on hepatic levels of iNOS and COX-II. (**A**) Hepatic levels of inducible nitric oxide synthase (iNOS); (**B**) relative quantitation of iNOS mRNA expression (fold change relative to GAPDH); (**C**) hepatic levels of cyclooxygenase-2 (COX-II); (**D**) relative quantitation of COX-II mRNA expression (fold change relative to GAPDH. Data are expressed as mean ± SD (*n* = 4). APAP: acetaminophen; NAC: N-acetyl cysteine; Prist: Pristimerin; GAPDH: glyceraldehyde-3-phosphate dehydrogenase. * *p* < 0.05, ** *p* < 0.01, *** *p* < 0.001, and **** *p* < 0.0001 using one-way ANOVA followed by the Tukey–Kramer multiple comparisons post hoc test.

As revealed in Figure 5, APAP administration led to a marked (*p* < 0.0001) rise in the hepatic level (Figure 5A,C) and mRNA expression (Figure 5B,D) of iNOS and COX-II, respectively. On the other hand, Prist (0.8 mg/kg) pretreatment markedly attenuated the mRNA expression and hepatic levels of iNOS and COX-II, where it restored the expression of iNOS to baseline levels.

**Figure 6:**
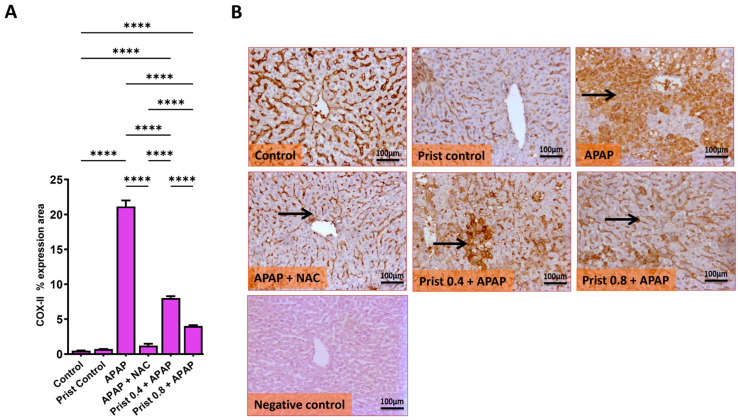
Effect of Prist on hepatic immune expression levels of COX-II. (**A**) bar graph showing the % expression of COX-II; (**B**) COX-II immunohistochemical study. Data are expressed as mean ± SD (*n* = 4). APAP: acetaminophen; NAC: N-acetyl cysteine; Prist: Pristimerin; GAPDH: glyceraldehyde-3-phosphate dehydrogenase. **** *p* < 0.0001 using one-way ANOVA followed by the Tukey–Kramer multiple comparisons post hoc test.

As revealed in Figure 6, APAP administration significantly enhanced the immune expression of COX-II (Figure 6A,B), where the immune stain of COX-II in the control and Prist groups showed negative immunostaining in hepatocytes. Meanwhile, the APAP group showed extensive immunostained hepatocytes with marked cytoplasmic and nuclear staining. In addition, the NAC-pretreated group showed negative to faint immunopositive-stained hepatocytes. The Prist 0.4-pretreated group showed focal, few to minimal positive immunostained hepatocytes, and the Prist 0.8-pretreated group showed scattered immunopositive-stained hepatocytes and cytoplasmic staining of positive hepatocytes (Figure 6B). Assessment of percent expression area of COX-II (Figure 6A) showed a significant (*p* < 0.05) increase in COX-II expression in the APAP-injected group versus the control group. The pretreatment with Prist (0.8 mg/kg) significantly (*p* < 0.0001) lowered the COX-II expression compared to the APAP-injected group.

### 3.5. Prist Restrained APAP-Induced Activation of NFκB and Downstream Cytokines

**Figure 7:**
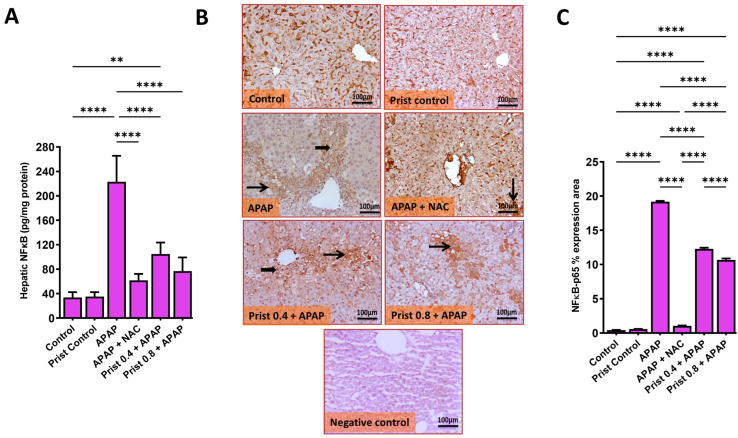
Effect of Prist on the expression of NF-κB in hepatic tissue. (**A**) Hepatic levels of nuclear factor kappa B (NF-κB); (**B**) nuclear factor kappa-B p65 subunit (NFκB-p65) immunohistochemical study; (**C**) bar graph showing the % expression of NFκB-p65. Data are expressed as mean ± SD (*n* = 4). APAP: acetaminophen; NAC: N-acetyl cysteine; Prist: Pristimerin. ** *p* < 0.01, and **** *p* < 0.0001 using one-way ANOVA followed by the Tukey–Kramer multiple comparisons post hoc test.

As presented in Figure 7, APAP administration resulted in a considerable (*p* < 0.0001) rise in the hepatic level of NFκB (Figure 7A) and elevated immunoexpression of NFκB-p65 in the hepatic tissue compared to control mice. Prist (0.8 mg/kg) administration alleviated the elevated levels of NFκB and repressed its activation compared to the APAP group, where representative IHC of NFκB-p65 expression in hepatic sections revealed negative to faint immunopositive-stained hepatocytes in the control and Prist control groups. Meanwhile, the APAP-injected group showed multifocal to coalescing immunostained hepatocytes with positively stained inflammatory cells, and high cytoplasmic staining with few nuclear-stained inflammatory cells and hepatocytes. On the other hand, the NAC-treated group showed few immunopositive-stained hepatocytes. The Prist 0.4-pretreated group showed focal to coalescing faint-stained hepatocytes and inflammatory cells, and the Prist 0.8-pretreated group showed focal minimal to mild immunostained hepatocytes, with cytoplasmic expression in positive hepatocytes. Assessment of percent expression area of NFκB-p65 (Figure 7B) showed a significant (*p* < 0.05) elevation in NFκB-p65 expression in the APAP-injected group compared with the control group. The pretreatment with Prist (0.8 mg/kg) significantly (*p* < 0.0001) lowered the NFκB-p65 expression versus the APAP-injected group.

**Figure 8:**
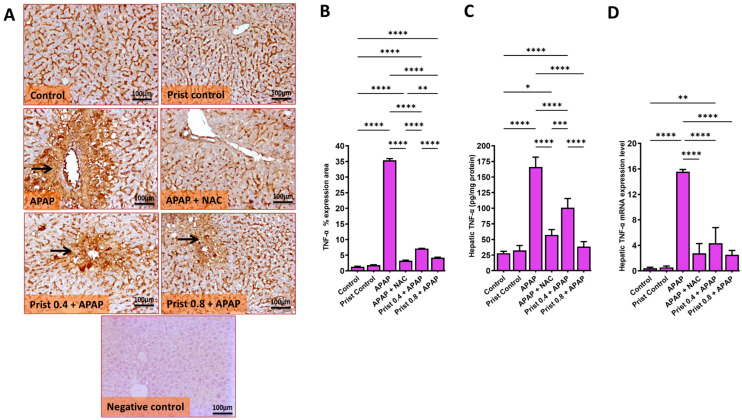
Effect of APAP on the markers of inflammation in hepatic tissue. (**A**) Tumor necrosis factor-α (TNF-α) immunohistochemical study; (**B**) bar graph showing the % expression of TNF-α; (**C**) hepatic levels of TNF-α; (**D**) relative quantitation of TNF-α mRNA expression (fold change relative to GAPDH). Data are expressed as mean ± SD (*n* = 4). APAP: acetaminophen; NAC: N-acetyl cysteine; Prist: Pristimerin; GAPDH: glyceraldehyde-3-phosphate dehydrogenase. * *p* < 0.05, ** *p* < 0.01, *** *p* < 0.001, and **** *p* < 0.0001 using one-way ANOVA followed by the Tukey–Kramer multiple comparisons post hoc test.

As revealed in Figure 8, the APAP injection resulted in a marked (*p* < 0.0001) elevation in the immune (Figure 8A,B), protein (Figure 8C), and mRNA expression levels (Figure 8D) of TNF-α; on the other hand, Prist (0.8 mg/kg) pretreatment significantly (*p* < 0.05) attenuated the mRNA expression and hepatic levels of TNF-α. Additionally, representative IHC of TNF-α expression in the control and Prist control groups showed negative immunostaining in hepatocytes. Meanwhile, the APAP-injected group showed extensive immunostained hepatocytes with marked cytoplasmic and nuclear staining. In addition, the NAC-treated group revealed a negative to faint immunopositive-stained hepatocytes, and Prist 0.4-pretreated group showed focal mild immunostained hepatocyte and cytoplasmic with nuclear expression in positive hepatocytes. The Prist 0.8-pretreated group showed focal faint-stained hepatocytes and inflammatory cells (Figure 8A).

Assessment of percentage expression area of TNF-α (Figure 8B) showed a significant (*p* < 0.0001) increase in TNF-α expression in the APAP-injected group versus the control group. The pretreatment with Prist (0.8 mg/kg) significantly (*p* < 0.05) decreased the TNF-α expression versus the APAP-injected group.

**Figure 9:**
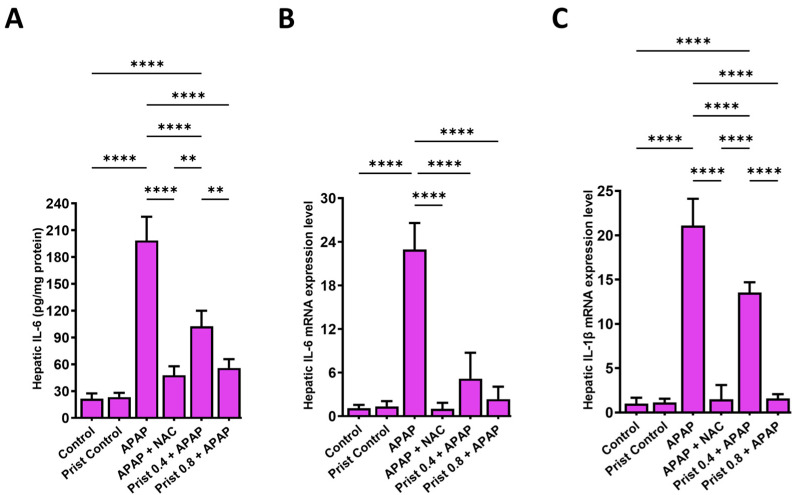
Effect of APAP on the markers of inflammation in hepatic tissue. (**A**) Hepatic levels of interleukin-6 (IL-6); (**B**) relative quantitation of IL-6 mRNA expression (fold change relative to GAPDH); (**C**) relative quantitation of interleukin-1β (IL-1β) mRNA expression (fold change relative to GAPDH). Data are expressed as mean ± SD (*n* = 4). APAP: acetaminophen; NAC: N-acetyl cysteine; Prist: Pristimerin; GAPDH: glyceraldehyde-3-phosphate dehydrogenase. ** *p* < 0.01, and **** *p* < 0.0001 using one-way ANOVA followed by the Tukey–Kramer multiple comparisons post hoc test.

In addition, the hepatic level of IL-6 (Figure 9A) and mRNA expression levels of IL-6 and IL-1β (Figure 9B,C) were significantly increased (*p* < 0.0001) in the APAP-injected group versus the control group, whereas administration of Prist (0.8 mg/kg) significantly (*p* < 0.05) lowered the hepatic levels and mRNA expression of IL-6 and mRNA expression of IL-1β in comparison with the APAP-administered group.

### 3.6. Prist Ameliorated APAP-Induced Change in p-PI3K and p-AKT Expression in Hepatic Tissues

**Figure 10:**
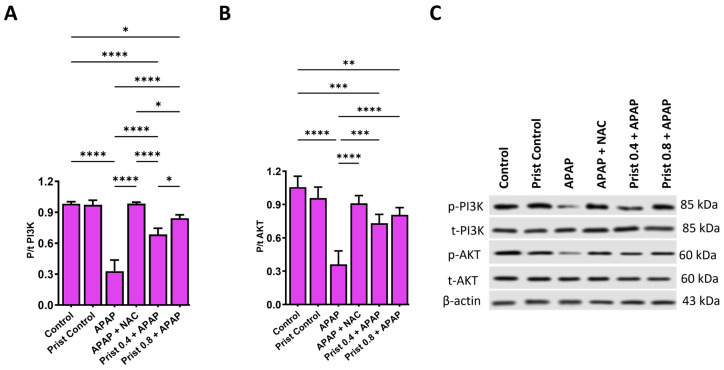
Effect of Prist on liver expression of p-PI3K and p-AKT. Bar graph showing protein expression of (**A**) p-PI3K/t-PI3K and (**B**) p-AKT/t-AKT. (**C**) Representative images of Western blot analysis of p-PI3K/t-PI3K and p-AKT/t-AKT in the liver of each group of rats. Data are expressed as mean ± SD (*n* = 4). APAP: acetaminophen; NAC: N-acetyl cysteine; Prist: Pristimerin; AKT: protein kinase B; PI3K: phosphoinositide 3-kinase. * *p* < 0.05, ** *p* < 0.01, *** *p* < 0.001, and **** *p* < 0.0001 using one-way ANOVA followed by the Tukey–Kramer multiple comparisons post hoc test.

Hepatic p-PI3K and p-AKT protein expression were markedly decreased (*p* < 0.0001) in the APAP group versus control mice. p-PI3K and p-AKT expression were significantly (*p* < 0.05) elevated in the Prist 0.4-, Prist 0.8-, and NAC-administered groups (Figure 10).

### 3.7. Prist Attenuated APAP-Induced Apoptosis in Hepatic Tissue

**Figure 11:**
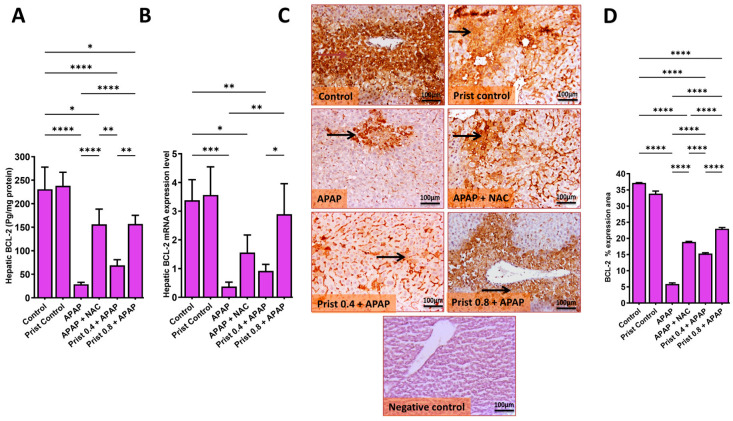
Effect of Prist on hepatic levels of B-cell leukemia/lymphoma-2 (BCL-2). (**A**) Hepatic levels of BCL-2; (**B**) relative quantitation of BCL-2 mRNA expression (fold change relative to GAPDH); (**C**) BCL-2 immunohistochemical study; (**D**) bar graph showing the % expression of BCL-2. Data are expressed as mean ± SD (*n* = 4). APAP: acetaminophen; NAC: N-acetyl cysteine; Prist: Pristimerin; GAPDH: glyceraldehyde-3-phosphate dehydrogenase. * *p* < 0.05, ** *p* < 0.01, *** *p* < 0.001, and **** *p* < 0.0001 using one-way ANOVA followed by the Tukey–Kramer multiple comparisons post hoc test.

As shown in Figure 11, the hepatic level (Figure 11A), mRNA (Figure 11B), and immunoexpression (Figure 11C) of the anti-apoptotic protein BCL-2 were significantly (*p* < 0.0001) decreased in the APAP-injected group versus the control group. This level was significantly (*p* < 0.05) increased in the Prist 0.8-pretreated group versus the APAP-injected mice, as representative IHC of BCL-2 expression showed coalescing perivascular extensive immunostained hepatocytes and cytoplasmic staining of positive hepatocytes in the control group, and diffuse intense expression in hepatocytes in the Prist control group. Meanwhile, the APAP-injected group showed focal, minimal to mild immunopositive-stained hepatocytes. On the other hand, the NAC-treated group showed moderate to high cytoplasmic immunostaining of hepatocyte; the Prist 0.4-pretreated group showed moderate to high immunopositive expression of BCL2 in hepatocytes; and the Prist 0.8-pretreated group showed high immunopositive-stained hepatocytes with intense cytoplasmic expression in positive hepatocytes. Assessment of percentage expression area of BCL-2 exhibited a significant (*p* < 0.05) rise in BCL2 expression in the APAP-injected group versus the normal mice. The pretreatment with Prist (0.8 mg/kg) significantly (*p* < 0.05) decreased the BCL-2 expression versus the APAP-injected group (Figure 11D)

**Figure 12:**
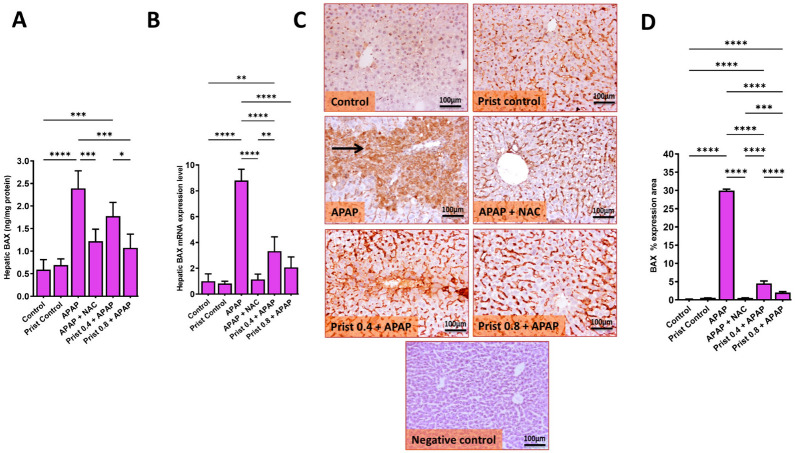
Effect of Prist on the hepatic level of BCL2-associated X (BAX). (**A**) Hepatic levels of BAX; (**B**) relative quantitation of BAX mRNA expression (fold change relative to GAPDH); (**C**) BAX immunohistochemical study; (**D**) bar graph showing the % expression of BAX. Data are expressed as mean ± SD (*n* = 4). APAP: acetaminophen; NAC: N-acetyl cysteine; Prist: Pristimerin; GAPDH: glyceraldehyde-3-phosphate dehydrogenase. * *p* < 0.05, ** *p* < 0.01, *** *p* < 0.001, and **** *p* < 0.0001 using one-way ANOVA followed by the Tukey–Kramer multiple comparisons post hoc test.

**Figure 13:**
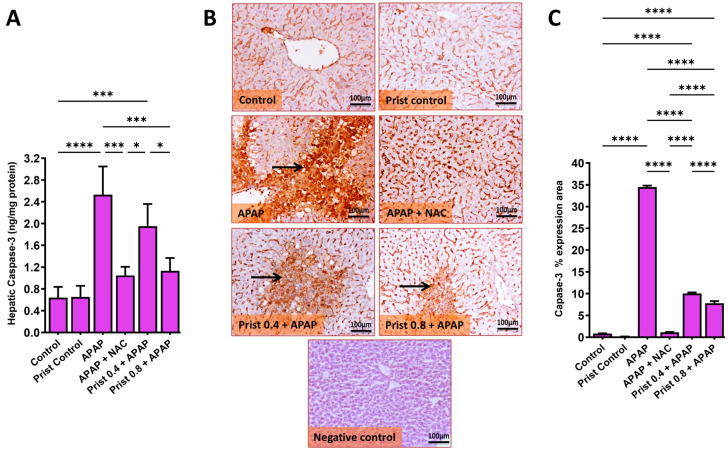
Effect of Prist on the hepatic level of caspase-3. (**A**) Hepatic levels of caspase-3; (**B**) caspase-3 immunohistochemical study; (**C**) bar graph showing the % expression of caspase-3. Data are expressed as mean ± SD (*n* = 4). APAP: acetaminophen; NAC: N-acetyl cysteine; Prist: Pristimerin; GAPDH: glyceraldehyde-3-phosphate dehydrogenase. * *p* < 0.05, *** *p* < 0.001, and **** *p* < 0.0001 using one-way ANOVA followed by the Tukey–Kramer multiple comparisons post hoc test.

On the other hand, the hepatic levels, mRNA, and immunoexpression of the apoptotic proteins BAX (Figure 12) and the hepatic levels and immunoexpression of caspase-3 (Figure 13) were significantly (*p* < 0.0001) raised in the APAP-administered group versus the control mice. Prist 0.8 pretreatment counteracted APAP-induced elevations in the levels and expression of these apoptotic indices, as Prist significantly attenuated the hepatic levels (Figure 12A), mRNA (Figure 12B), and immunoexpression (Figure 12C) of BAX, and hepatic levels (Figure 13A) and immunoexpression (Figure 13B) of capase-3.

As shown in Figure 12C and Figure 13B, respectively, representative IHC of BAX and caspase-3 expression in the hepatic sections of the control and Prist control groups showed negative immunostaining in hepatocytes, whereas the APAP-treated group showed multifocal to coalescing or diffuse extensive immunostained hepatocytes and in invading inflammatory cells, with marked cytoplasmic staining in swollen hepatocytes. On the other hand, the NAC-treated group showed negative to faint immunopositive-stained hepatocytes; the Prist 0.4-pretreated group showed mild to moderate focal area of immunostained hepatocytes; and the Prist 0.8-pretreated group showed minimal to mild faint expression in hepatocytes. Assessment of percentage expression area of BAX (Figure 12D) and caspase-3 (Figure 13C) revealed a significant (*p* < 0.0001) rise in BAX and capase-3 expression in the APAP-injected group versus the normal mice. The pretreatment with Prist (0.8 mg/kg) markedly (*p* < 0.05) decreased this expression area versus to the APAP- injected mice.

## 4. Discussion

APAP is the most widely consumed non-prescription analgesic-antipyretic medication [42]. Poisoning from APAP has emerged as a significant public health issue as it can lead to serious liver damage in the case of incorrect doses, intentional self-harm, or non-intentional overdoses [43]. The current study showed the protective impacts of Prist administration on APAP hepatotoxic changes and clarified its possible mechanisms. Administration of Prist prior to APAP successfully decreased APAP hepatotoxicity through antioxidant/anti-inflammatory, and anti-apoptotic pathways. Prist treatment endorsed hepatic antioxidants, suppressed pathogenic inflammatory pathways such as NF-κB/iNOS/COX-II/cytokines, and upregulated p-PI3K/p-AKT expression. Moreover, Prist pretreatment counteracted apoptotic regulators like BAX and caspase-3, and upregulated the anti-apoptotic BCL-2. Notably, Prist itself did not cause any adverse effects at the selected dosage.

Our data showed that APAP administration caused noticeable hepatocellular damage in the form of necrosis, inflammatory changes, and leucocytes infiltration. Consistently, there was a marked rise in hepatic transaminases, ALP, and GGT, as well as a decrease in albumin, which confirmed the hepatic injury [44]. These findings are in line with previous reports that clarified the deleterious effect of APAP overdose on the hepatic tissue [45,46]. Interestingly, Prist pretreatment ameliorated hepatocellular damage and inhibited the leakage of cytosolic enzymes, indicating the ability of Prist to conserve the cellular integrity.

APAP overdose primarily causes oxidative stress through the activation of mitochondrial superoxide, nitric oxide, and their reactive product peroxynitrite, which is the primary cause of oxidative and nitrosative stress [47]. This results in the depletion of antioxidants and hepatocellular disruption. In the present work, APAP markedly reduced hepatic GSH, GR, GST, TAC, and SOD activities, while it intensified the hepatic content of the lipid peroxidative biomarker MDA. However, Prist administration significantly enriched the antioxidants and inhibited lipid peroxidation. These results agree with the results of previous studies that showed the potent antioxidant properties of Prist [24,26,48].

Advanced inflammation resulting from oxidative burden and cellular necrosis is another significant element in the development of APAP hepatotoxicity [49]. The hepatic expression and activation of NF-κB, a stress-associated transcription factor that regulates genes implicated in the inflammatory response, oxidative stress, and cytoprotection, were found to be elevated by APAP overdose [50]. Under normal conditions, the inhibitory protein IκB-α prevents the translocation of NF-κB into the nuclei, causing NF-κB inactivation. Degradation of IκB-α occurs when macrophages are stimulated by inflammatory signals [51], where, after high dose of APAP, the activation of NF-κB leads to an increase in the levels of pro-inflammatory cytokines, including TNF-α, IL-6, and IL-1β [50,52,53]. In the present study, APAP administration significantly increased NF-κB, IL-1β, IL-6, TNF-α, and iNOS expression, while Prist pretreatment inhibited these increments. Our findings are supported by earlier studies that showed the potent ability of Prist to modulate NF-κB activation and subsequently to ameliorate the production of pro-inflammatory mediators in chronic obstructive pulmonary disease [54], LPS-induced inflammation [55], and doxorubicin-induced cardiotoxicity [56].

The PI3K/AKT pathway is a pivotal signaling pathway that mediates cell-survival, apoptosis, oxidative stress, necrosis, and autophagy. It plays a key role in the process of hepatic regeneration following damage [57]. Experimental evidence has demonstrated that the PI3K/AKT signaling pathway provides protection to the liver by either suppressing or increasing the expression of downstream target proteins [58]. This pathway can protect the liver through phosphorylating target proteins such as BCL-2 family proteins, Nrf2, and NF-κB to control hepatocyte apoptosis and liver damage [59,60]. Previous studies have documented that the activation of PI3K/AKT pathway results in the enhancement of anti-apoptotic proteins like BCL-2 and a suppression of pro-apoptotic proteins such as BAX, thus promoting cell survival [61,62]. Furthermore, the functions of anti-apoptotic proteins are endorsed by AKT. Hence, suppression of the PI3K/AKT pathway is linked to a change in the BAX/BCL-2 ratio, and thus, the enhancement of apoptosis [63,64]. In the present study, our results showed that APAP administration produced a significant lowering in p-PI3K and p-AKT levels, contributing to hepatocellular damage, in agreement with the previous studies which found that a high dose of APAP can induce liver injury via downregulation of p-PI3K and p-AKT proteins [65,66]. However, the Prist-pretreated animal groups showed a significant increase in both p-PI3K and p-AKT protein expression in a dose-dependent manner.

A new emerging body of evidence indicates the significance of hepatocyte apoptosis in the development of certain liver disorders, such as viral, cholestatic, and toxin-induced hepatic damage [67]. This phenomenon is especially intriguing in cases of toxic injury, such as overdose from APAP, where necrosis is the primary process of hepatocyte loss [53]. Many reports have documented the crosstalk between apoptosis and necrosis after high-dose APAP administration [68,69], and confirmed the correlation between APAP-induced apoptotic cell death and caspase-3 activation [69,70]. Caspases are one of the most precise markers of the apoptotic cell death, as they play a direct or indirect role in all dimensions of apoptotic signaling within the cell [71]. Proteins belonging to the BCL-2 family exhibit both pro- and anti-apoptotic properties [72]. In recent times, there has been a growing trend of quantifying BCL-2 levels as an anti-apoptotic marker in experimental APAP hepatotoxicity, as the majority of these studies indicate the decreased expression levels of BCL-2 with APAP toxicity [73]. BAX is a member of the pro-apoptotic BCL-2 family, mostly identified in the cytosol, where, in the presence of stress, BAX has the ability to go to the outer mitochondrial membrane [74], resulting in the formation of pores in the outer membrane that leak intermembrane proteins, thus facilitating apoptosis [74]. It is noteworthy that the translocation of BAX to the mitochondria was detected within 2 h following APAP overdose [75]. The outcomes of our study validated earlier observations by showing that APAP could significantly induce hepatocellular apoptosis. This was apparent from the increased expression and levels of BAX and caspase-3, accompanied by decreased expression of BCL-2. It is noteworthy that pretreatment with Prist noticeably ameliorated changes in these apoptotic markers generated by APAP. Our data were in harmony with the previous report of [24]. These results provide a reasonable basis to assume that the capacity of Prist pretreatment to regulate the BAX/BCL-2/caspase-3 and PI3K/AKT signaling pathways could inhibit hepatocyte apoptosis and result in the repair of hepatocellular damage.

Limitation of the study: The specificity of Prist’s effect on migrated monocyte-derived macrophages and resident macrophages—Kupffer cells—and the underlying cellular mechanisms has not been assessed in the present study and needs further elucidation, as there is ongoing debate on the macrophages’ involvement in the pathophysiology of APAP-induced liver damage. Because hepatic macrophages produce pro-inflammatory cytokines and mediators such as TNF-α and NO, it has been shown that they contribute to APAP-induced hepatotoxicity; however, hepatic macrophages also contribute to hepatoprotection by producing mediators and cytokines like IL-6 and IL-18-binding proteins that prevent inflammation [76,77]. It is still debatable how macrophages contribute to the pathophysiology of APAP-induced liver damage. The variability and/or adaptability of hepatic macrophages may account for their dual roles as pro-toxicant and hepatoprotective cells [76,77].

## 5. Conclusions

Prist administration significantly ameliorates APAP-induced acute liver injury by maintaining the hepatocellular oxidant/antioxidant balance, concurrent with a potent anti-inflammatory and anti-apoptotic microenvironment. These effects are mediated through the modulation of NF-κB/iNOS/COX-II downstream cytokines, PI3K/AKT, and BAX/BCL-2/caspase-3 signaling cascades.

## Figures and Tables

**Table 1:** Hepatic Histopathological Scores.

Score	Steatosis	Necrosis	Inflammation
0	None	None	None
1	Less than 5% of tissue affected	Minimal, few	1 focus/5 field examined
2	More than 5–50% of tissue affected	Mild to moderate, multifocal to coalescing necrotic area	2-3 foci/5 field examined
3	More than 50% of tissue exhibited steatosis	Diffuse, many	More than 3 foci/5 field examined

## Data Availability

The datasets produced throughout and/or examined throughout the existing study are available from the corresponding author upon reasonable request.
